# Deletion of lynx1 reduces the function of α6* nicotinic receptors

**DOI:** 10.1371/journal.pone.0188715

**Published:** 2017-12-05

**Authors:** Rell L. Parker, Heidi C. O’Neill, Beverley M. Henley, Charles R. Wageman, Ryan M. Drenan, Michael J. Marks, Julie M. Miwa, Sharon R. Grady, Henry A. Lester

**Affiliations:** 1 Division of Biology and Biological Engineering, California Institute of Technology, Pasadena, CA, United States of America; 2 Institute for Behavioral Genetics, University of Colorado Boulder, Boulder, CO, United States of America; 3 Department of Pharmacology, Northwestern University Feinberg School of Medicine, Chicago, IL, United States of America; 4 Department of Psychology and Neuroscience, University of Colorado, Boulder, CO, United States of America; 5 Department of Biological Sciences, Lehigh University, Bethlehem, PA, United States of America; University of Michigan, UNITED STATES

## Abstract

The α6 nicotinic acetylcholine receptor (nAChR) subunit is an attractive drug target for treating nicotine addiction because it is present at limited sites in the brain including the reward pathway. Lynx1 modulates several nAChR subtypes; lynx1-nAChR interaction sites could possibly provide drug targets. We found that dopaminergic cells from the substantia nigra pars compacta (SNc) express lynx1 mRNA transcripts and, as assessed by co-immunoprecipitation, α6 receptors form stable complexes with lynx1 protein, although co-transfection with lynx1 did not affect nicotine-induced currents from cell lines transfected with α6 and β2. To test whether lynx1 is important for the function of α6 nAChRs *in vivo*, we bred transgenic mice carrying a hypersensitive mutation in the α6 nAChR subunit (α6L9′S) with lynx1 knockout mice, providing a selective probe of the effects of lynx1 on α6* nAChRs. Lynx1 removal reduced the α6 component of nicotine-mediated rubidium efflux and dopamine (DA) release from synaptosomal preparations with no effect on numbers of α6β2 binding sites, indicating that lynx1 is functionally important for α6* nAChR activity. No effects of lynx1 removal were detected on nicotine-induced currents in slices from SNc, suggesting that lynx1 affects presynaptic α6* nAChR function more than somatic function. In the absence of agonist, lynx1 removal did not alter DA release in dorsal striatum as measured by fast scan cyclic voltammetry. Lynx1 removal affected some behaviors, including a novel-environment assay and nicotine-stimulated locomotion. Trends in 24-hour home-cage behavior were also suggestive of an effect of lynx1 removal. Conditioned place preference for nicotine was not affected by lynx1 removal. The results show that some functional and behavioral aspects of α6-nAChRs are modulated by lynx1.

## Introduction

Nicotinic acetylcholine receptors (nAChRs) are essential for many aspects of normal brain function, but their most important public health relevance is their role in nicotine addiction. Nicotine addiction causes approximately 12% of premature worldwide deaths in people over 30 years of age (WHO Global Report: Mortality Attributable to Tobacco). This pervasive harm makes it imperative to find more effective methods of nicotine cessation. In the search for a pharmacological therapy to aid in nicotine cessation, one particular subclass of nAChRs, those containing the α6 subunit, has garnered intense interest as a drug target [1, 2]. Studies have shown that nAChRs containing the α6 subunit (α6* nAChRs) are necessary for the rewarding effects of nicotine [3–5]. The α6* nAChR is localized to the dopaminergic (DA) neurons of the ventral tegmental area (VTA) and substantia nigra pars compacta (SNc), as well as neurons of the locus coeruleus, retinal ganglia, superior colliculi (SC) and medial habenula (MHb) [6–9]. This localization of α6* nAChR to just a few neuronal cell types suggests that drugs targeting this specific subtype would have fewer side effects than drugs targeting nAChRs with more widespread expression in brain [2, 3, 10]. Because of the difficulty of finding small molecule drugs that selectively target the α6* nAChR, it might be useful to investigate interactions of this receptor with other proteins. Such studies may identify other useful drug target sites.

Previous studies demonstrate that lynx1 is capable of modulating several classes of nicotinic receptors, including α4β2 [11, 12], α7 [13–15] and α3β4(α5) [16] nAChR subtypes. Lynx1 can act as a brake on nicotinic receptor function by causing a shift to the right of concentration-response curves in α4β2 nAChRs, shifting receptor subunit stoichiometry by affecting assembly in the endoplasmic reticulum [17], increasing the rate of desensitization and slowing the recovery from desensitization [11–13, 18], as well as influencing plasticity and spine dynamics [19]. However, no previous studies examined whether lynx1 produces effects on α6* nAChR function, although a water-soluble variant of lynx1 forms complexes with α6* nAChRs [20].

To facilitate studies of the α6 nicotinic receptor subunit in a mouse model, a hypersensitive mutation has been introduced in the pore lining M2 domain (L9′S mutation: the Leu 9′ residue in the M2 domain was mutated to Ser)[21]. Mice containing the α6L9′S mutation are BAC transgenic mice that express several copies of the α6 gene modified by a L9′S mutation [21]. This L9′S mutation produces hypersensitive receptors that are sensitive to lower nicotine concentrations, demonstrated with a shift to the left in dose-response relations [22–24]. This strategy of generating mice with hypersensitive nAChRs enables investigation of behavioral traits at nicotine doses that activate only the hypersensitive subtypes as well as more precise biochemical and physiological assays [23]. Mice with α6L9′S nicotinic receptors exhibit several phenotypes resulting from over-activation of α6* nAChRs in DA neurons of the VTA and SNc, including locomotor hyperactivity and augmented DA release [21]. Several subsequent studies have used these mice to investigate the role of the α6* nAChR function *in vivo* [25–28]. For example, the α4 nAChR subunit was deleted from the α6L9′S mice by cross breeding [27], with the resulting line of mice showing considerably attenuated effect of the hypersensitive mutation, indicating that the receptor subtype, (α4β2)(α6β2)β3, is important for the expression of hyperactive phenotypes.

We chose to investigate whether lynx1 regulates α6* nAChR expression and function. For the current study, we used the α6L9′S mice to determine whether deletion of lynx1 affects α6* nAChRs, either by reduction or augmentation of the known phenotypes of the α6L9′S mice. We cross bred the two mouse lines, α6L9′S and lynx1 null mutant (lynx1KO), and utilized biochemical approaches, along with electrophysiology and behavior in mice with both lynx1KO and α6L9′S mutations to determine whether lynx1 regulates α6* nAChRs. We found effects of lynx1 on some α6* nAChR-mediated functional assays and on some mouse behaviors; and we found that lynx1 directly interacts with α6* nAChRs, providing a possible mechanistic basis for the functional and behavioral effects.

## Results

### Protein and mRNA levels and interactions

**Fig 1:**
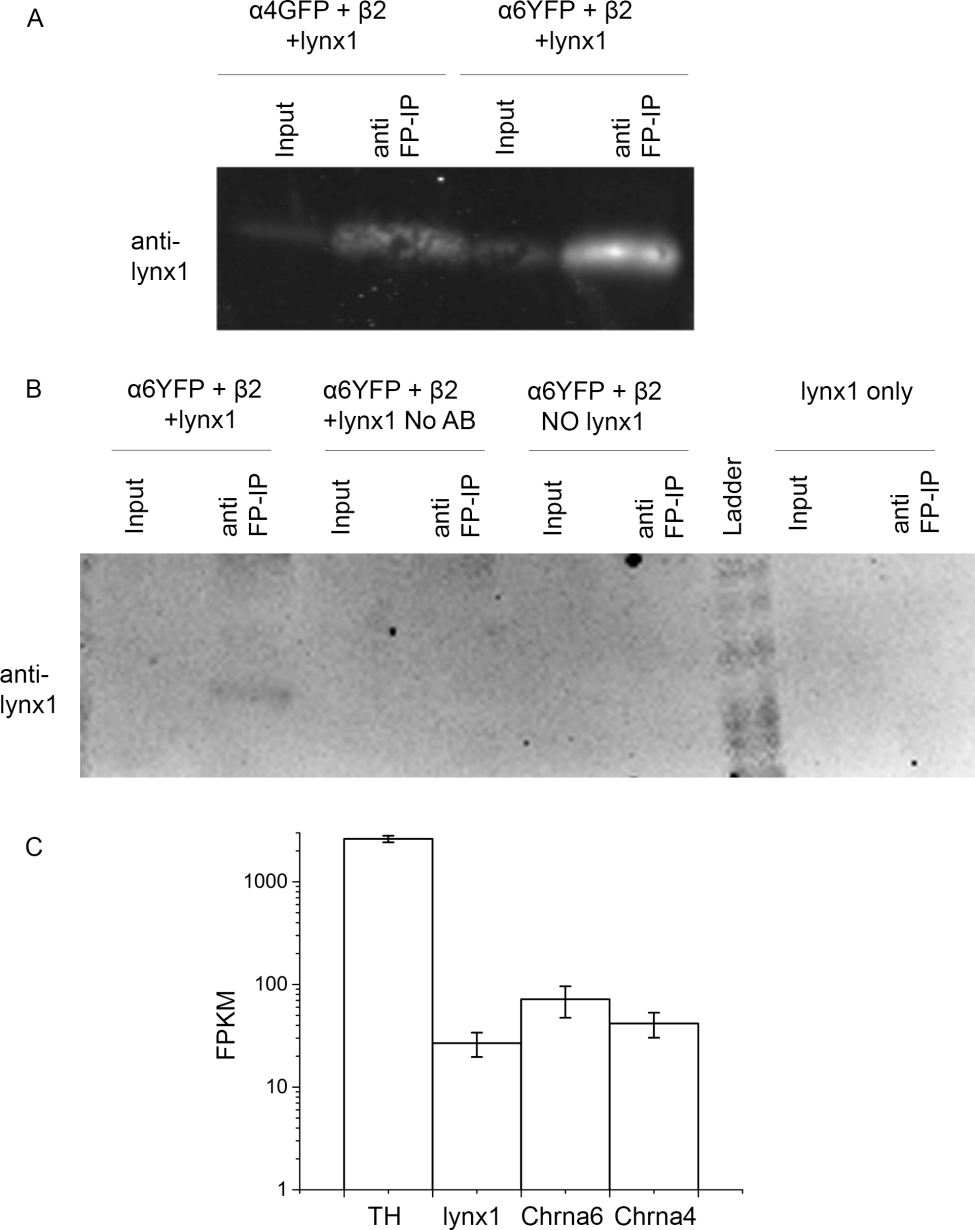
A) Western blot of cells transfected with either α4GFP, β2, and lynx1 (left two lanes) or α6YFP, β2, and lynx1 (right two lanes) and subjected to immunoprecipitation. Input lanes (first and third lanes) are cell extracts. The immunoprecipitation was performed using protein A beads coated with anti-GFP antibody (termed anti-FP); the blot was performed with an anti-lynx1 antibody. B) Western blot of cells transfected with either α6YFP, β2, and lynx1 (left 4 lanes), α6YFP and β2 without lynx1 1 (lanes 5 and 6), or lynx1 only (right two lanes). In the 3^rd^ and 4^th^ lanes, no anti-GFP antibody was added to the immunoprecipitation extract. The blot was performed with an anti-lynx1 antibody. C) The lynx1 gene is expressed in DA neurons (in addition to *TH* (tyrosine hydroxylase), *Chrna6* (α6 nicotinic receptor), and *Chrna4* (α4 nicotinic receptor). We collected and pooled 20 SNc neurons (n = 3 pools) using laser-capture microscopy, then assessed transcriptome-wide expression by RNA-Seq. Cufflinks was used to calculate the (FPKM) expression levels in laser captured SNc neurons. *TH* is expressed at 2615 ± 194.7 FPKM; *Lynx1* is expressed at 26.79 ± 7.12, *Chrna6* is expressed at 71.67 ± 24.31 FPKM, and *Chrna4* is expressed at 41.67 ± 11.39 FPKM (n = 3 pools). These data are presented on a log10 scale. FPKM: fragments per kilobase per million mapped reads.

In order to test whether lynx1 interacts with α6* nicotinic receptors, we conducted a co-immunoprecipitation experiment using HEK-293 cells transfected with cDNA encoding nAChRs and lynx1. HEK-293 cells were transiently transfected with either α4GFP or α6YFP nicotinic subunits, plus β2WT nicotinic subunits, and lynx1. We used an anti-GFP antibody to pull down the α6YFP or the α4GFP fusion protein using conditions that retain stable protein complexes. Because GFP and YFP differ by only a few amino acids at regions distinct from epitopes, the two fluorophores are precipitated well by anti-GFP antibodies. We analyzed the immunoprecipitated nAChR complexes by western blot analysis using an anti-lynx1 antibody (Fig 1A). In material from cells transfected with lynx1 plus either α4GFP and β2WT or α6YFP and β2WT, we detected lynx1 on the blot, indicating that lynx1 forms a stable complex with α4β2 and α6β2 nAChRs. These data confirmed previous reports that lynx1 does immunoprecipitate with α4β2 nicotinic receptors [11]. In control experiments, when either lynx1 or the nAChR subunits were omitted or the anti-GFP antibody was not added, no lynx1 was detected Fig 1B). This indicates that lynx1 binds in a complex with α6YFPβ2 receptors as well as with α4GFPβ2 nAChRs.

We used RNA-Seq to confirm that lynx1 RNA transcript is present in the brain regions that are associated with α6 nicotinic receptors. SNc cells were identified by anatomical location in midbrain slices and pools of 20 cells were collected using laser capture microdissection and pooled (n = 3 pools). RNA-Seq was performed and the relative levels of lynx1, α4 (*Chrna4*), and α6 (*Chrna6*) were measured, in addition to tyrosine hydroxylase (*TH*), a gene highly and specifically expressed in DA neurons, which served as a positive control (Fig 1C). This shows that lynx1 is indeed present in DA neurons, along with α6 and α4 nAChR subunits. Because > 90% of DA neurons express α6 subunit protein [6], it is highly likely that lynx1 is present in the same SNc DA neurons as α6 subunits.

We next determined whether there is a functional significance to the interaction between lynx1 and α6* nAChRs. Wildtype α6* nAChRs have previously shown much weaker agonist-induced currents than other nAChR subtypes in most heterologous systems [29–32]. By transfecting α6YFP and β2WT nicotinic receptor subunits with and without lynx1 in HEK293 cells, we tested whether addition of lynx1 would increase the surface expression and functional activity of α6* nicotinic receptors. Using whole cell patch clamp electrophysiology, responses to a puff of 300 μM nicotine were recorded. Cells were selected for patching only if fluorescence was visually evident, suggesting that α6YFP protein was present in the cell. In the case of transfection with α6YFP + β2, n = 12 cells were patched and puffed with nicotine. None showed a nicotine-induced current > 10 pA. When lynx1 was transfected in addition to α6YFPβ2WT, there was again little or no nicotine response. In this case, n = 7 cells were puffed with nicotine and none showed a response above 10 pA. Therefore, in HEK293 cells, lynx1 by itself does not appear to enable expression of functional α6β2-nAChRs.

**Fig 2:**
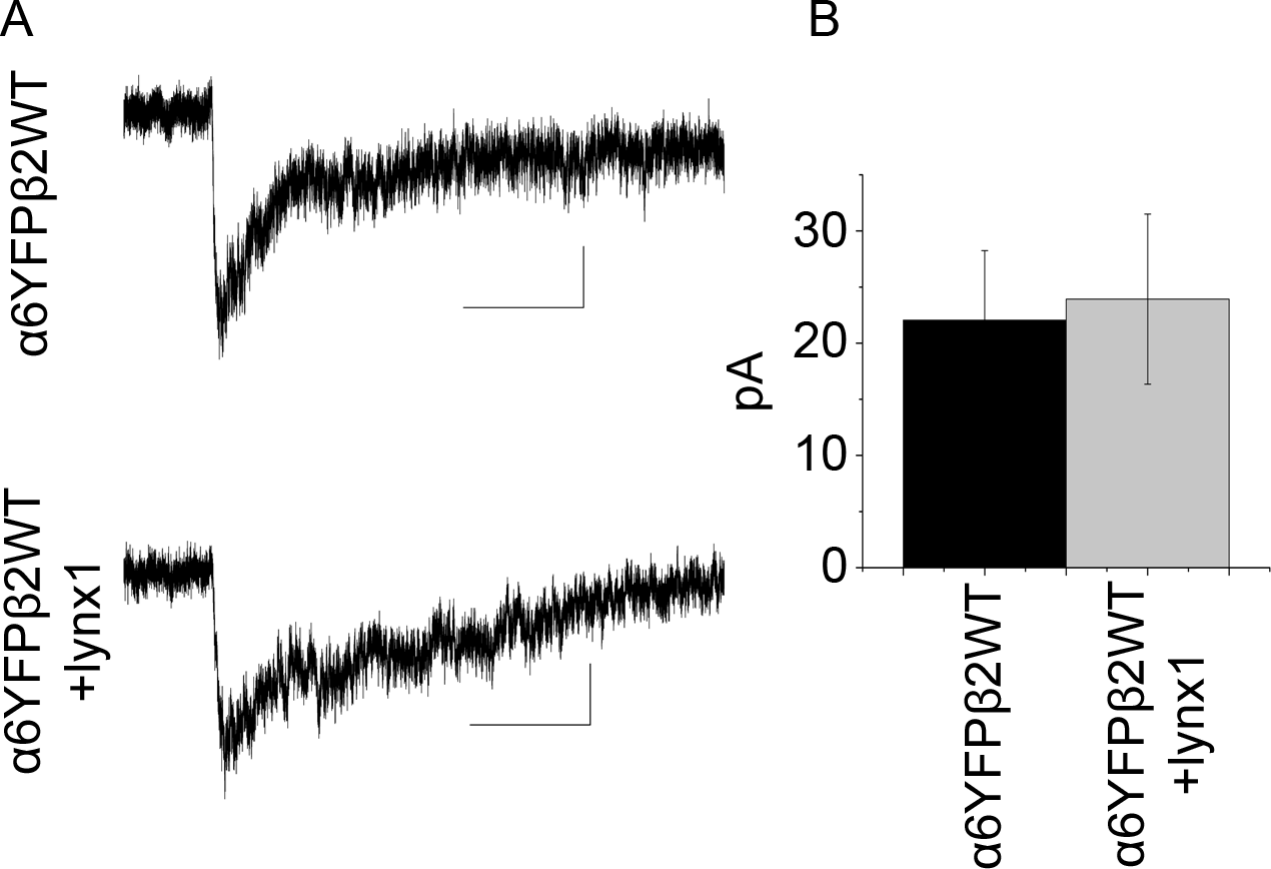
A) Average whole-cell nicotine-induced currents (200 ms puffs, 300 μM) from N2a cells transfected with either α6YFP and β2 (8 cells) or α6YFP, β2, and lynx1 (6 cells). Scale is 1 s and 5 pA for both traces. B) Graph showing average peak response; error bars are SEM. Average response for α6YFP and β2 was 22.0 ± 6.2 pA and the average response for α6YFP, β2, and lynx1 was 23.9 ± 7.6 pA. There was no significant effect of lynx1 addition.

N2a cells do express functional α6β2-nAChR on the surface with modest efficiency [32, 33]. Therefore, we used N2a cells to determine whether adding lynx1 changes the efficiency of functional α6β2-nAChR expression. In the case of α6YFPβ2WT, 8 of 10 cells exhibited a response to 300 μM nicotine (average peak response = 22.0 ± 6.2 pA). When cells were transfected with α6YFPβ2WT + lynx1, 6 of 9 cells responded (23.9 ± 7.6 pA). Average traces are shown in Fig 2A. Adding lynx1 did not significantly affect the size of the response (Fig 2B) or the percentage of cells responding. The addition of lynx1 had no major effect on the response waveform.

Neurons *in vivo* have mechanisms for more efficient functional expression of various nAChRs; possibly these mechanisms are crucial for α6* nAChRs [6–8, 34]. Previous studies have used mice containing hypersensitive α6* nAChRs to isolate and amplify the α6* nAChR responses [21]. α6* receptors are expressed in the DA neurons and visual system axons, and the α6L9′S mutation unmasks α6* nAChR function to varying degrees in those regions [21]. To study the effects of lynx1 on α6* nAChRs, we bred the α6L9′S mice to lynx1KO mice [13, 21]. We hypothesized that the effects of lynx1 on the hypersensitive α6* nAChRs may resolve changes in α6* function resulting from lynx1KO.

To determine whether lynx1KO affected the quantity of nAChRs, we measured [^125^I]epibatidine binding to membrane preparations from several brain regions of four genotypes of mice (lynx1 WT and KO on both α6WT and α6L9′S background). To assess changes in selected nAChRs subunit populations, we analyzed the amount of [^125^I]epibatidine binding that was inhibited by the addition of α-conotoxin MII (α-CtxMII)(50 nM), which selectively blocks α6β2* sites, or addition of a low concentration (50 nM) of cytisine, which selectively blocks the α4β2* binding sites. Regions measured included striatum (ST), olfactory tubercle (OT), superior colliculus (SC), assayed for both sites, as well as frontal cortex (fCX), hippocampus (HP) and visual cortex (vCX) assayed for only α4β2 sites. We assayed mice from each genotype; results are presented in Tables 1 and 2. Overall, little or no difference was seen when lynx1 was knocked out. No significant differences were found between lynx1WT and KO on either α6 background in any region for α6β2* binding sites. In ST, the lynx1KO compared to lynx1WT on the α6WT background exhibited a modest but significant increase in α4β2 binding sites of ~ 15% (*p* < 0.05). In SC, a modest but significant decrease (18%) in α4β2* binding sites was noted for the lynx1KO compared to the lynx1WT on the α6L9′S background (*p* < 0.01). No other differences were detected in any of the six regions for these sites.

**Table 1:** α6β2 binding sites in isolated membrane preparations.

	ST	OT	SC
	% of control		
lynx1WT/ α6WT	100 ± 10.7 (n = 14)	100 ± 12.4 (n = 13)	100 ± 8.7 (n = 12)
lynx1KO/ α6WT	130.1 ± 24.1 (n = 12)	105.4 ± 17.7 (n = 7)	114.2 ± 14.6 (n = 9)
lynx1WT/ α6L9′S	115.9 ±11.4 (n = 17)	94.4 ± 14.1 (n = 13)	75.0 ± 5.8 (n = 15)
lynx1KO/ α6L9′S	84.7 ± 14.9 (n = 8)	104.6 ± 14.8 (n = 8)	61.8 ± 17.3 (n = 8)

Data expressed as % of lynx1WT/α6WT. Data were analyzed for effect of lynx1 on each α6 background for each region by t-test. No significant effects of the lynx1KO genotype were found.

**Table 2:** α4β2 binding sites in isolated membrane preparations.

	ST	OT	SC	fCX	vCX	HP
	% of control					
lynx1WT/ α6WT	100 ± 2.7 (n = 13)	100 ± 2.1 (n = 13)	100 ± 2.5 (n = 12)	100 ± 13.9 (n = 7)	100 ± 5.3 (n = 7)	100 ± 5.0 (n = 6)
lynx1KO/ α6WT	115.1 ± 6.0* (n = 12)	103.9 ± 2.3 (n = 10)	106.8 ± 4.8 (n = 9)	101.8 ± 13.0 (n = 5)	108.8 ± 10.3 (n = 5)	116.9 ± 7.2 (n = 5)
lynx1WT/ α6L9′S	110.9 ± 2.5 (n = 17)	111.3 ± 3.7 (n = 16)	103.7 ± 3.4 (n = 14)	101.7 ± 5.7 (n = 6)	97.9 ± 5.3 (n = 7)	111.0 ± 11.1 (n = 8)
lynx1KO/ α6L9′S	115.3 ± 2.5 (n = 9)	111.7 ± 2.6 (n = 7)	85.4 ±3.5** (n = 7)	114.8 ± 11.8 (n = 4)	100.0 ± 8.6 (n = 5)	131.4 ± 8.9 (n = 5)

Data expressed as % of lynx1WT/α6WT. Data analyzed for effects of lynx1 on each α6 background for each region by t-test. Significant effects of lynx1 are seen for ST for the lynx1WT/α6WT and lynx1KO/α6WT genotypes (* P<0.05) and for SC with lynx1WT/α6L9′S and lynx1KO/α6L9′S (** P<0.01).

To better assess the α6β2* binding sites in small regions, we used quantitative autoradiography with [^125^I]epibatidine, with and without 50 nM α-CtxMII in 10 brain regions from the lynx1WT and KO mice (n = 5–7 mice per group) on the α6L9′S background. These data (Table 3) confirm that lynx1 has little or no effect on numbers of α6β2 binding sites in α6L9′S mice. Data presented in Table 4 show binding that was not inhibited by α-CtxMII, which represents mostly α4β2 sites. No significant differences were found in any of the 10 regions assayed. Although epibatidine binding does not differentiate between surface and subcellular localization of nicotinic receptors, these data do indicate that, in general, nicotinic receptor expression is not affected by lynx1KO. Previous studies evaluating epibatidine binding in the α6L9′S vs WT mice ST and OT have shown either no change in α-CtxMII-sensitive or -resistant binding in the ST or OT [27], or a slight increase in α-CtxMII-sensitive in OT and no change in α-CtxMII-resistant binding [21]. Because the epibatidine binding experiments have shown that neither the α6L9′S nor the lynx1KO produced marked change in number of α6β2 binding sites, we hypothesized that the lynx1KO x α6L9′S mouse would be useful in analyzing effects of lynx1KO on the function of α6* receptors in the mouse brain.

**Table 3:** α6β2 binding sites by autoradiography.

	NAc	OT	ST	opt	OPN
	fmol of binding /mg wet weight of tissue				
lynx1WT/ α6L9′S	0.76 ± 0.25 (n = 6)	0.79 ± 0.16 (n = 6)	0.88 ± 0.19 (n = 6)	1.17 ± 0.44 (n = 6)	4.38 ± 0.52 (n = 6)
lynx1KO/ α6L9′S	1.22 ± 0.17 (n = 7)	1.02 ± 0.21 (n = 7)	0.91 ± 0.10 (n = 7)	1.47 ± 0.31 (n = 7)	4.29 ± 1.45 (n = 6)
	DLG	VLG	SN	VTA	SC
lynx1WT/ α6L9′S	5.15 ± 0.76 (n = 6)	4.42 ± 1.18 (n = 6)	1.58 ± 0.26 (n = 5)	3.06 ± 1.32 (n = 5)	5.41 ± 0.51 (n = 6)
lynx1KO/ α6L9′S	6.48 ± 1.88 (n = 6)	3.14 ± 1.49 (n = 6)	1.65 ± 0.42 (n = 5)	1.83 ± 1.09 (n = 5)	6.73 ±1.48 (n = 7)

Data are expressed as fmol of binding /mg wet weight of tissue. Region abbreviations are: NAc, nucleus accumbens; OT, olfactory tubercle; ST, striatum; opt, optic tracts; OPN, olivary pretectal nucleus; DLG, dorsal lateral geniculate; VLG, ventral lateral geniculate; SN, substantia nigra; VTA, ventral tegmental area; SC superior colliculus. Data were analyzed for effect of lynx1 for each region by t-test. No significant effects of lynx1 were found.

**Table 4:** α4β2 binding sites by autoradiography.

	NAc	OT	ST	opt	OPN
	fmol of binding /mg wet weight of tissue				
lynx1WT/ α6L9′S	2.59 ± 0.14 (n = 6)	2.15 ± 0.16 (n = 6)	3.49 ± 0.21 (n = 6)	2.97 ± 0.21 (n = 6)	7.60 ± 0.53 (n = 6)
lynx1KO/ α6L9′S	2.20 ± 0.22 (n = 7)	2.13 ± 0.17 (n = 7)	3.70 ± 0.43 (n = 7)	2.88 ± 0.24 (n = 7)	8.82 ± 0.82 (n = 6)
	DLG	VLG	SN	VTA	SC
lynx1WT/ α6L9′S	12.35 ± 0.98 (n = 6)	8.06 ± 0.35 (n = 6)	8.13 ± 0.26 (n = 5)	8.24 ± 0.67 (n = 5)	7.83 ± 0.39 (n = 6)
lynx1KO/ α6L9′S	12.05 ± 1.31 (n = 6)	8.91 ± 0.85 (n = 6)	8.31± 0.60 (n = 5)	8.06 ± 0.95 (n = 5)	7.44 ± 0.73 (n = 7)

Data are expressed as fmol of binding /mg wet weight of tissue. Region abbreviations are: NAc, nucleus accumbens; OT, olfactory tubercle; ST, striatum; opt, optic tracts; OPN, olivary pretectal nucleus; DLG, dorsal lateral geniculate; VLG, ventral lateral geniculate; SN, substantia nigra; VTA, ventral tegmental area; SC superior colliculus. Data were analyzed for effect of lynx1 for each region by t-test. No significant effects of lynx1 were found.

### Functional measurements in several brain regions: ^86^Rb^+^ efflux

**Fig 3:**
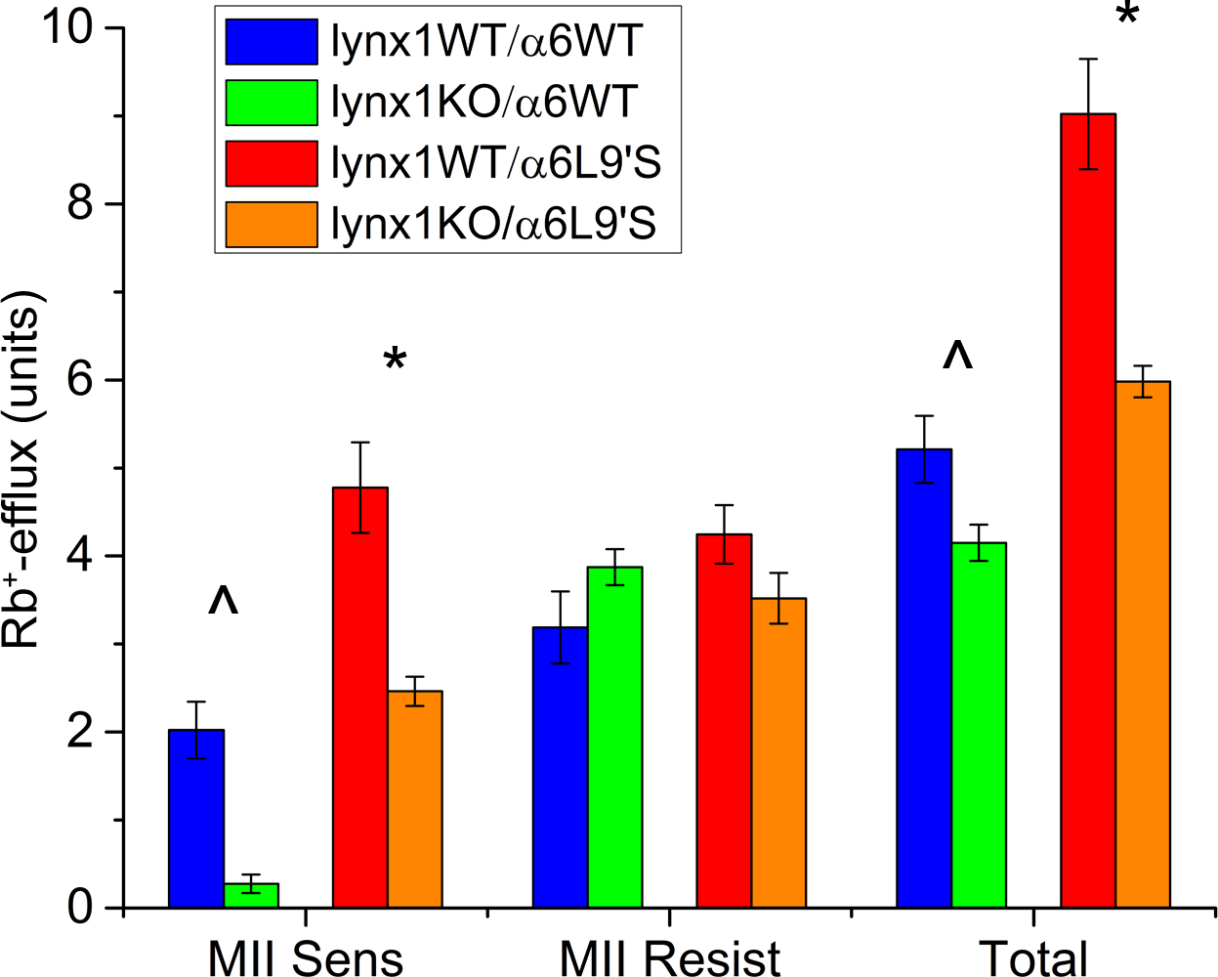
^86^Rb^+^ efflux evoked by 3 μM nicotine in superior colliculus synaptosomal preparations. Animal numbers: lynx1WT/α6WT = 7, lynx1KO/α6WT = 7, lynx1WT/α6L9′S = 7, lynx1KO/α6L9′S = 5. Left panel: α-CtxMII-sensitive efflux, mediated via α6β2* nicotinic receptor (total efflux, minus efflux in the presence of α-CtxMII). Lynx1WT/α6WT and lynx1KO/α6WT are significantly different (*p* < 0.001), designated by ^. Lynx1WT/α6L9′S and lynx1KO/α6L9′S are significantly different (*p =* 0.005), designated by *. Middle panel: α-CtxMI-Iresistant efflux, mediated by α4β2* nicotinic receptors (efflux in the presence of α-CtxMII). There are no significant differences between lynx1WT/α6WT and lynx1KO/α6WT or between lynx1WT/α6L9′S and lynx1KO/α6L9′S. Right panel: total ^86^Rb^+^ efflux in superior colliculus. Lynx1WT/α6WT and lynx1KO/α6WT are significantly different (*p =* 0.032), designated by ^. Lynx1WT/α6L9′S and lynx1KO/α6L9′S are significantly different (*p =* 0.003), designated by *.

To measure changes in the agonist-induced flux through nAChRs, we used ^86^Rb^+^ efflux measurements in synaptosomal preparations from SC, HP, fCX and vCX. In the SC we observed a significant decrease in the α-CtxMII-sensitive ^86^Rb^+^ efflux in lynx1KO/α6WT mice compared to lynx1WT/α6WT mice, as well as in the lynx1KO/α6L9′S mice compared to the lynx1WT/α6L9′S (Fig 3). No change was found for α-CtxMII-resistant nAChR function. These results suggest that there is less α6* receptor function on the surface of SC cells when lynx1 is absent in mice, either with wildtype α6 subunits or with the α6L9′S mutation. We found no differences in HP, fCX or vCX (Table 5); these regions contain no measurable α6*-nAChRs. The decrease observed in α6* receptor function in SC indicates that lynx1 is necessary for normal level of function of α6* nicotinic receptors in this region. Perhaps fewer receptors are retained on the surface without lynx1, or an interaction of lynx1 with α6β2* nAChRs facilitates function by changing ratios of subtypes containing α6 subunits.

**Table 5:** ^86^Rb^+^ efflux from crude synaptosomal preparations of HP, vCX, and fCX, for each genotype.

[nicotine], μM	0.5 μM	50 μM	0.5 μM	50 μM
hippocampus (HP)	lynx1WT/α6WT	lynx1WT/α6WT	lynx1WT/α6L9′S	lynx1WT/α6L9′S
	1.34 ± 0.17 (7)	5.05 ± 0.24 (7)	1.72 ± 0.27 (6)	5.19 ± 0.49 (6)
	lynx1KO/α6WT	lynx1KO/α6WT	lynx1KO/α6L9′S	lynx1KO/α6L9′S
	1.39 ± 0.14 (5)	5.62 ± 0.73 (5)	0.95 ± 0.13 (5)	5.34 ± 0.53 (5)
visual cortex (vCX)	lynx1WT/α6WT	lynx1WT/α6WT	lynx1WT/α6L9′S	lynx1WT/α6L9′S
	1.65 ± 0.12 (7)	8.25 ± 0.46 (7)	1.58 ± 0.23 (6)	7.49 ± 0.72 (6)
	lynx1KO/α6WT	lynx1KO/α6WT	lynx1KO/α6L9′S	lynx1KO/α6L9′S
	1.46 ± 0.22 (5)	6.70 ± 0.62 (5)	1.56 ± 0.23 (5)	6.93 ± 0.72 (5)
frontal cortex (fCX)	lynx1WT/α6WT	lynx1WT/α6WT	lynx1WT/α6L9′S	lynx1WT/α6L9′S
	1.75 ± 0.25 (7)	6.84 ± 0.36 (7)	1.79 ± 0.12 (6)	7.38 ± 0.73 (6)
	lynx1WT/α6WT	lynx1WT/α6WT	lynx1WT/α6L9′S	lynx1WT/α6L9′S
	1.57 ± 0.30 (5)	6.77 ± 0.56 (5)	2.13 ± 0.35 (5)	6.65 ± 0.89 (5)

Data are normalized to baseline and given as mean ± sem (n). No significant differences among lynx1 or α6 genotypes were noted.

### Dopamine neurons: Functional and biochemical measurements

**Fig 4:**
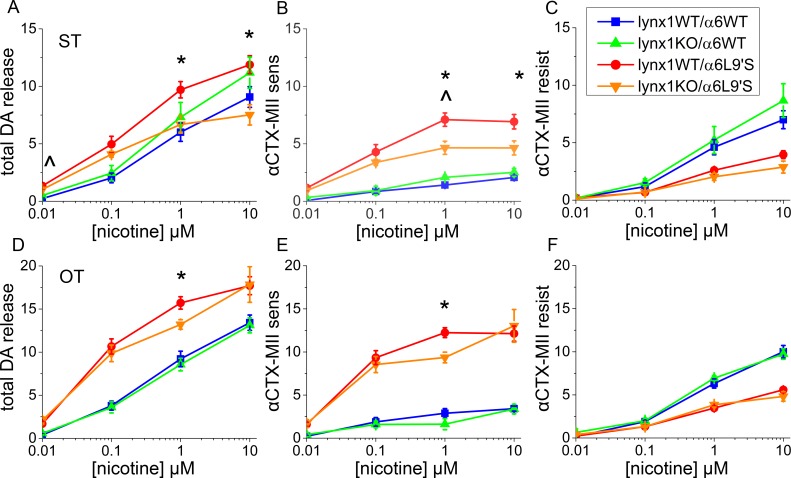
Nicotine-induced DA release from synaptosomal preparations. Animal numbers: lynx1WT/α6WT = 6, lynx1KO/α6WT = 4, lynx1WT/α6L9′S = 7, lynx1KO/α6L9′S = 4. A) Total nicotine-induced DA release from striatal (ST) synaptosomes. Lynx1WT/α6WT and lynx1KO/α6WT differ significantly at 0.01 μM nicotine (*p =* 0.031), designated by ^. Lynx1WT/α6L9′S and lynx1KO/α6L9′S differ significantly at 1 μM (*p =* 0.023) and 10 μM (*p =* 0.024) nicotine, designated by *. B) α-CtxMII-sensitive nicotine-mediated DA release from ST synaptosomes. Lynx1WT/α6WT and lynx1KO/α6WT differ significantly at 1 μM nicotine (*p =* 0.015). Lynx1WT/α6L9′S and lynx1KO/α6L9′S differ significantly at 1 μM (*p =* 0.022) and 10 μM (*p =* 0.042) nicotine. C) α-CtxMII-resistant nicotine-mediated DA release from ST synaptosomes. There were no significant differences between lynx1WT/α6WT and lynx1KO/α6WT or between lynx1WT/α6L9′S and lynx1KO/α6L9′S. D) Total nicotine-mediated DA release from olfactory tubercle (OT) synaptosomes. Lynx1WT/α6WT and lynx1KO/α6WT are not significantly different from each other. lynx1WT/α6L9′S and lynx1KO/α6L9′S differ significantly at 1 μM nicotine (*p =* 0.042). E) α-CtxMII-sensitive nicotine-mediated DA release from OT synaptosomes. Lynx1WT/α6WT and lynx1KO/α6WT do not differ significantly. Lynx1WT/α6L9′S and lynx1KO/α6L9′S differ significantly at 1 μM nicotine (*p =* 0.010). F) α-CtxMII-resistant nicotine-mediated DA release from OT synaptosomes. There were no significant differences between lynx1WT/α6WT and lynx1KO/α6WT or between lynx1WT/α6L9′S and lynx1KO/α6L9′S.

We performed three different measurements on DA neurons to characterize changes at the cellular and synaptic level. To probe possible changes due to lynx1 KO at the nerve terminals of DA neurons, synaptosomal preparations from ST and OT were used to measure nicotine-mediated DA release. Data for ST are shown in Fig 4A–4C, and data for OT are shown in Fig 4D–4F. Previous studies established that the α6L9′S mice have a larger α-CtxMII-inhibited component (α6β2* nAChR) of nicotine-mediated DA release, with a complementary reduction of α-CtxMII-resistant (α4β2* nAChR) nicotine-mediated DA release [21]. The α6L9′S nicotine-mediated DA release concentration response curve is also shifted to the left: the synaptosomes are sensitive to lower concentrations of nicotine. In this set of experiments, the lynx1WT α6L9′S mice results were similar to those reported previously for both ST and OT, showing increased α-CtxMII-sensitive activity (α6β2*) and decreased α-CtxMII-resistant activity (α4β2*). Notably, ST of the lynx1KO/α6L9′S mice showed an intermediate response, with lynx1KO/α6L9′S mice exhibiting a decreased proportion of α-CtxMII-sensitive receptor-mediated response (Fig 4B). This effect was less robust in OT and was not seen in either region with α6WT mice. The absence of lynx1 had no detectable effect on the α4β2*-mediated DA release in the ST or OT (Fig 4C and 4F). The reduction of α6* nicotinic receptor function in the absence of lynx1 in ST is similar to the pattern seen for SC ^86^Rb^+^ efflux (Fig 3). However, unlike the SC pattern, the lynx1KO/α6WT were not different from lynx1WT/α6WT. These data indicate that the modulatory effect of lynx1 on α6* nicotinic receptor can influence DA release, but the altered pattern may indicate some difference in subunit composition of the α6* nAChR populations or the level of lynx1 influence in the regions assayed.

**Fig 5:**
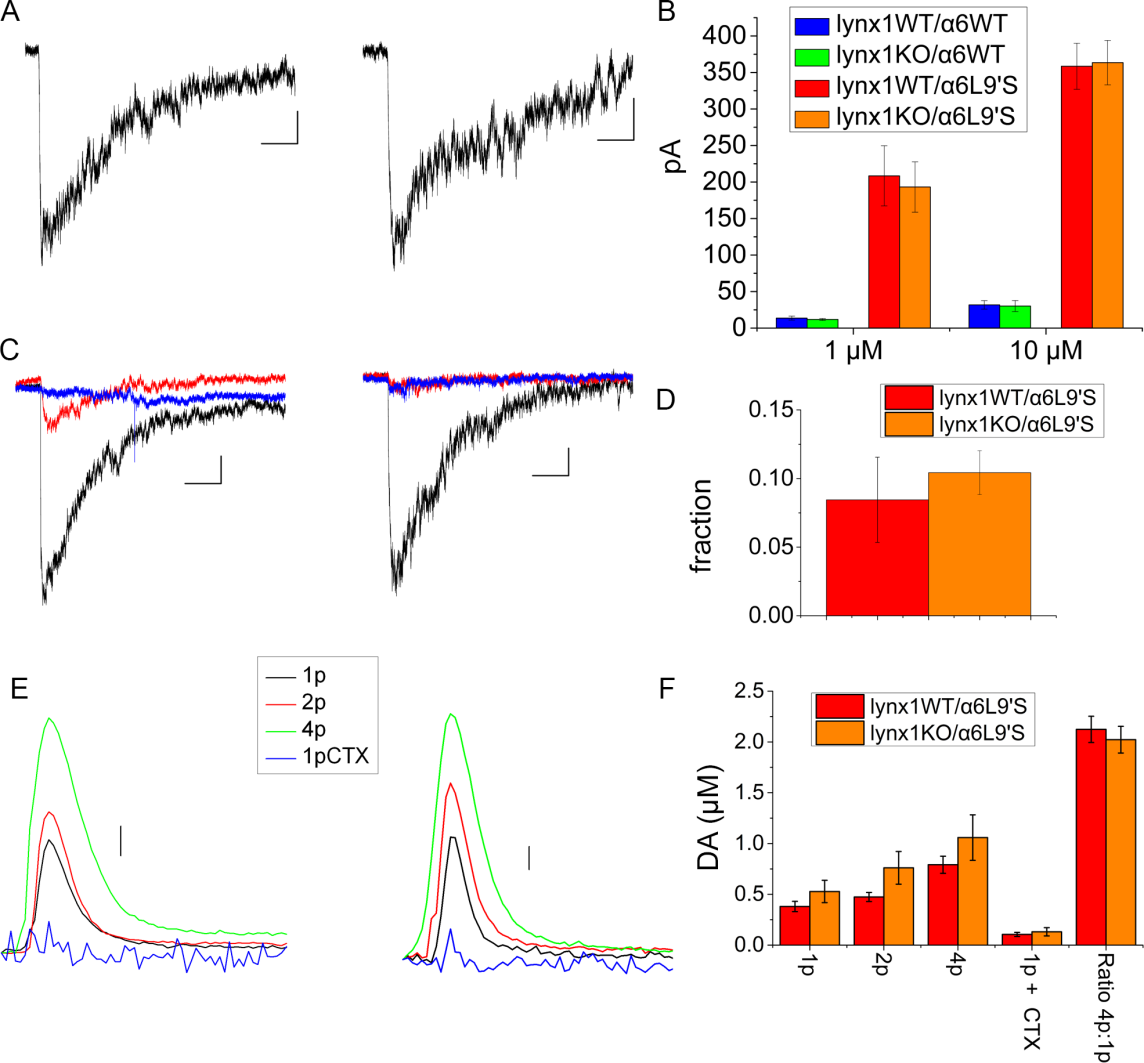
A) Representative whole-cell nicotine-induced currents from SNc of lynx1WT/α6L9′S and lynx1KO/α6L9′S mouse brain slices (puffs of 10 μM nicotine). Left is lynx1WT/α6L9′S; right is lynx1KO/α6L9′S. The scale is 2 s and 50 pA. B) Average peak currents induced by 1 and 10 μM nicotine, including lynx1WT/α6WT, lynx1KO/α6WT, lynx1WT/α6L9′S, and lynx1KO/α6L9′S mice. Values for 1 μM nicotine: lynx1WT/α6WT 13.6 ± 2.7 pA (7 cells), lynx1KO/α6WT 11.8 ± 1.4 pA (5 cells), lynx1WT/α6L9′S 208.5 ± 41.3 pA (11 cells), lynx1KO/α6L9′S 193.2 ± 34.4 pA (15 cells). Values for 10 μM nicotine: lynx1WT/α6WT 31.7 ± 5.9 pA (7 cells), lynx1KO/α6WT 30.2 ± 7.5 pA (5 cells), lynx1WT/α6L9′S 358.5 ± 31.6 pA (12 cells), lynx1KO/α6L9′S 363.5 ± 30.4 (17 cells). No significant differences were found between lynx1WT/α6WT and lynx1KO/α6WT or between lynx1WT/α6L9′S and lynx1KO/α6L9′S. C) Currents induced by 1 μM nicotine puffs before and after application of α-CtxMII. Response to 1 μM nicotine is black trace; red trace is 5 minutes after starting the flow of 100 μM α-CtxMII; blue trace is after 10 minutes of α-CtxMII. The left panel is lynx1WT/α6L9′S; the right panel is lynx1KO/α6L9′S. The scale is 2 s and 50 pA. D) Average fraction of signal remaining after application of 100 μM α-CtxMII. For lynx1WT/α6L9′S the percent remaining is 0.08 ± 0.03% (2 cells). For lynx1KO/α6L9′S the percent remaining is 0.10 ± 0.02% (3 cells). There is no significant difference between the two genotypes. E) Average DA release in response to various stimulations, as measured with FSCV. The left panel is lynx1WT/α6L9′S; the right panel is lynx1KO/α6L9′S. The scale is 0.1 μM DA. F) Average peak DA response. The values for lynx1WT/α6L9′S in μM DA are for 1p 0.38 ± 0.05, for 2p 0.47 ± 0.04, for 4p 0.79 ± 0.08, and for 1p + α-CtxMII 0.10 ± 0.02. The values for lynx1KO/α6L9′S in μM DA are for 1p 0.53 ± 0.11, for 2p 0.76 ± 0.16, for 4p 1.05 ± 0.22, and for 1p + α-CtxMII 0.13 ± 0.04. For the lynx1WT/α6L9′S the ratio of 4p:1p was 2.12 ± 0.13 and for the lynx1KO/α6L9′S the ratio of 4p:1p was 2.02 ± 0.13. For lynx1WT/α6L9′S there were 4 animals, 6 recording sites, except for the α-CtxMII studies that used 2 sites in 2 different animals. For lynx1KOα6L9′S there were 5 animals, 9 recording sites, except for the α-CtxMII studies that used 4 sites in 4 different animals. There were no significant differences between the two genotypes.

To assess function of α6* nAChR on the somata of DA neurons, in the presence and absence of lynx1, we recorded from the SNc of mouse brain slices. SNc DA neurons were identified, and a whole cell patch clamp configuration was obtained. We tested cells for I_h_ in voltage clamp and for firing patterns in current clamp to confirm that they were DA neurons [21]. DA cells were puffed with 1 and 10 μM nicotine (Fig 5A and 5B)), at intervals of > 4 min to allow recovery from desensitization. For some cells, 100 nM α-CtxMII was perfused in the bath to block the α6* component of the nicotinic response (Fig 5C). We found no difference between lynx1WT/α6WT and lynx1KO/α6WT animals in the currents induced by 1 or 10 μM nicotine (Fig 5B). However, as previously published, the α6L9′S mouse did show an increased response to nicotine compared to mice with wildtype α6 nAChRs [21]. Lynx1 deletion in lynx1KO did not affect the size of the response to nicotine in the α6L9′S mice (Fig 5B). Adding α-CtxMII to the bath blocked most of the response in the lynx1WT/α6L9′S and the lynx1KO/α6L9′S animals (Fig 5C). There was no significant difference in the fractional block by α-CtxMII in the lynx1KO/α6L9′S and the lynx1WT/α6L9′S (Fig 5D). This indicates that lynx1 removal does not affect the percentage of the signal contributed by α6* nAChRs in SNc.

As an additional method of assessing function at DA terminals, we performed fast scan cyclic voltammetry (FSCV) experiments in the dorsal striatum (Fig 5E). Previous studies indicate that α6L9′S mouse has altered DA release measured in the dorsal striatum [25, 27]. However, lynx1KO did not affect the electrically-stimulated DA release. We measured DA release in the lynx1WT/α6L9′S and lynx1KO/α6L9′S mice using single pulse (1p), 2 pulse (2p) or 4 pulse (4p) stimulations at 100 Hz. We also measured the α-CtxMII sensitivity of the 1p stimulation using bath application of 100 nM α-CtxMII. Average traces and the average peak responses are shown in Fig 5E and 5F. We also compared the ratio of 4p:1p peak response (Fig 4F), and there were no significant differences when lynx1 was deleted. For the lynx1WT/α6L9′S the ratio of 4p:1p was 2.12 ± 0.13, and for the lynx1KO/α6L9′S the ratio of 4p:1p was 2.02 ± 0.13. To compare the rate of DA uptake we fitted a single exponential decay to each waveform. There was no difference in the decay rate constant (τ) in the lynx1KO slices. Thus, in response to electrical stimulation, FSCV measurements reveal that the lynx1WT/α6L9′S mice and the lynx1KO/α6L9′S animals have similar peak DA release and τ.

The three DA neuron-based functional and biochemical measurements thus provide a mixed picture of lynx1 KO effects. In DA terminals of ST, we found decreased α6* specific nicotine-mediated DA release in lynx1KO/α6L9′S mice compared to lynx1WT/α6L9′S mice (Fig 4B). However, in SNc somata, deletion of lync1 did not change nicotine-induced currents (Fig 5A and 5B). In the absence of nicotine, we found no effects of lynx1 KO on electrically stimulated DA release using FSCV. Possible reasons for these differences may include processes that differ at the DA terminals vs the cell body. It is known that there are differences in the percentage of α6* receptors that reach the plasma membrane in terminals versus the cell body [1, 35], and effects of lynx1 removal may differentially affect the organelles in these regions [17]. Furthermore, the DA-release studies performed in striatal synaptosomes were evoked by nicotine and were concentration-dependent, whereas the FSCV studies were performed in the absence of exogenous ligand using electrical stimulation at 100Hz. Under the latter conditions, multiple processes may occur and the resulting DA release may be modified by some of these other effects of electrical stimulation. Therefore, it is unsurprising that detection of the effects of lynx1 on function of DA neurons, and on the role of their α6* nAChRs, depends on the method used and on other conditions of the assay.

### Nicotine-independent locomotor behavior

**Fig 6:**
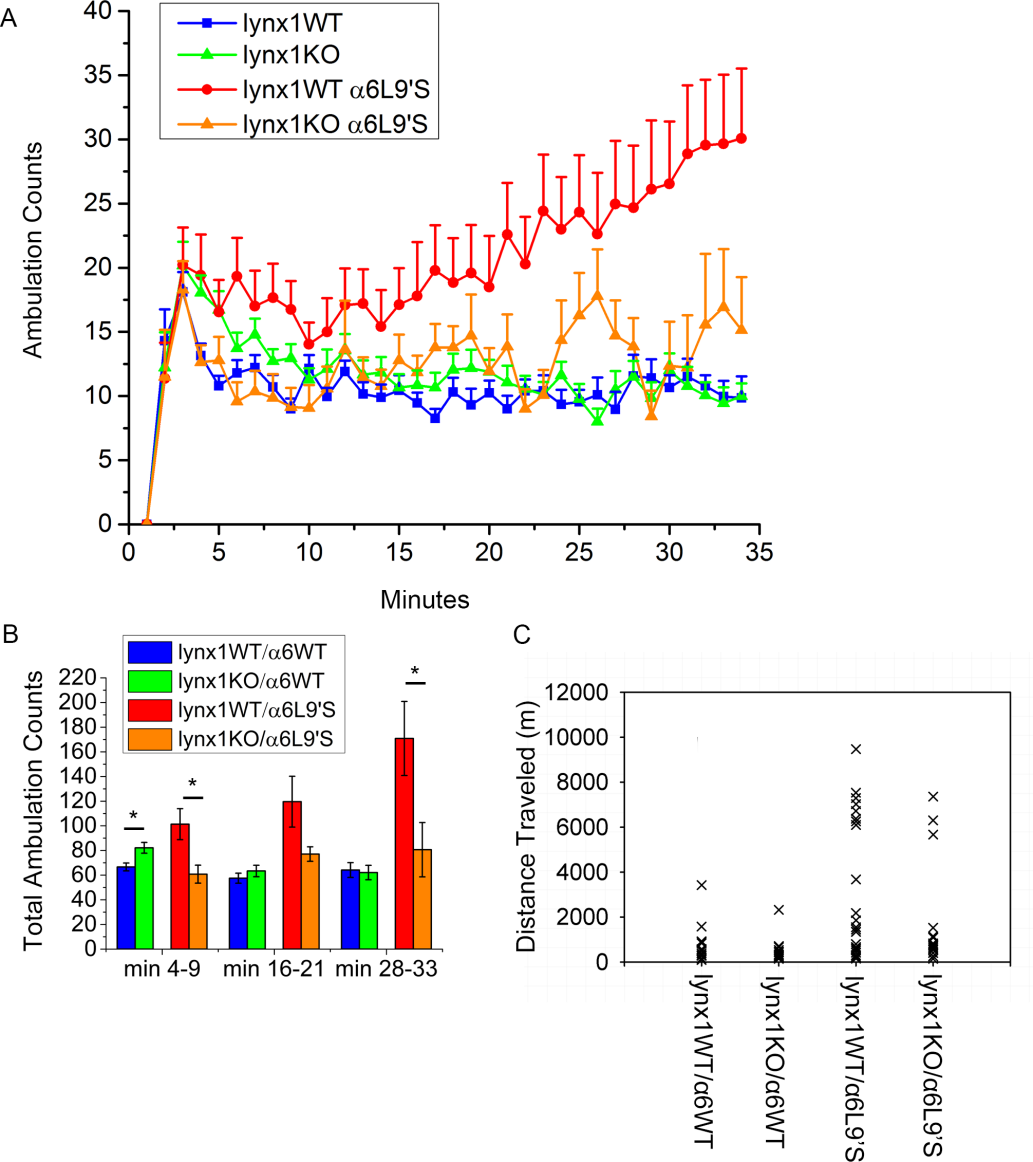
A) Response of mice to novel environment indicated by ambulation counts. Mice were placed in novel cages and their movement was measured by infrared beam breaks. Animal numbers: 20 lynx1WT/α6WT, 18 lynx1KO/α6WT, 24 lynx1WT/α6L9′S, 14 lynx1KO/α6L9′S. B) Sum of ambulation counts, with the experiment divided into 6 min bins to compare the ambulation during the beginning, middle, and end of the experiment. For lynx1WT/α6WT the values are: min 4–9 66.7 ± 3.2 counts, min 16–21 57.5 ± 4.1 counts, min 28–33 64.1 ± 6 counts. For lynx1KO/α6WT the values are: min 4–9 82.2 ± 4.4 counts, min 16–21 68.4 ± 4.7 counts, min 28–33 62.1 ± 5.9 counts. lynx1WT/α6WT and lynx1KO/α6WT were significantly different from each other in minutes 4–9 (*p =* 0.006). For lynx1WT/α6L9′S the values are: min 4–9 101.3 ± 12.6 counts, min 16–21 119.6 ± 20.6 counts, min 28–33 170.8 ± 30.0 counts. For lynx1KO/α6L9′S the values are: min 4–9 60.8 ± 7.3 counts, min 16–21 77.1 ± 10.0 counts, min 28–33 80.6 ± 22.0 counts. Lynx1WT/α6L9′S and lynx1KO/α6L9′S were significantly different from each other in min 4–9 (*p =* 0.026) and minutes 28–33 (*p =* 0.043). C) Total distance traveled over 24 h, as measured by automated mouse behavior analysis (AMBA). Animal numbers: 17 lynx1WT/α6WT, 17 lynx1KO/α6WT, 24 lynx1WT/α6L9′S, and 20 lynx1KO/α6L9′S. No statistical significance was found between lynx1WT/α6WT and lynx1KO/α6WT, or between lynx1WT/α6L9′S and lynx1KO/α6L9′S.

Next, we evaluated behavioral phenotypes to determine whether lynx1KO had effects on behaviors seen in α6L9′S mice. The α6L9′S behaviors include striking hyperactivity, with some mice running 2–10 km in a 24 h period [21, 27, 36]. We first tested habituation during the first 33 min in a novel environment. Fig 6A shows ambulations during this period. All the mice showed initially higher activity peaking at 2–3 min. As expected the lynx1WT/α6L9′S mice do not habituate appreciably. Of note, removal of lynx1 from the hyperactive mice significantly decreased their activity during minutes 4–9 (*p =* 0.026) and minutes 28–33 (*p =* 0.043) (Fig 6B). Lynx1WT/α6WT and lynx1KO/α6WT were significantly different from each other during minutes 4–9 only (*p =* 0.006) (Fig 6B).

In addition to the inability to habituate to a novel environment, some lynx1WT/α6L9′S mice are hyperactive during their active (dark) period [21, 27]. This occurs in 35–60% of animals of both sexes [21, 27]. We analyzed video recordings to ascertain the distance each mouse traveled during a 24-h home cage trial (Fig 6C). If hyperactivity is defined as movement greater than 1000 m in a 24-h period, 2 of 17 (12%) lynx1WT/α6WT mice and 1 of 17 (6%) lynx1KO/α6WT were hyperactive in this test, whereas, 14 of 24 (58.3%) of lynx1WT/α6L9′S mice in this cohort were hyperactive while 7 of 20 (35%) lynx1KO/α6L9′S showed hyperactivity. Using a more stringent cutoff of travelling 3000 m per 24-h period, 1 of 17 (6%) lynx1WT/α6WT mice and 0 of 17 (0%) lynx1KO/α6WT mice were hyperactive, while 9 of 24 (37.5%) lynx1WT/α6L9′S mice and only 3 of 20 (15%) lynx1KO/α6L9′S mice showed hyperactivity. Because there are two discrete populations and there is a low frequency in one of the groups, we applied Fisher’s exact test to determine whether lynx1KO was a factor in these differences on the L9′S background for night-time activity. The p-value was 0.14 for a cutoff of 1000, and 0.17 for a cutoff of 3000; these were not statistically significant differences. Activity behaviors in α6L9′S mice are not completely penetrant; only some of the mice exhibit the hyperactive behavior [21, 27, 36]. This wide phenotypic variation may have reduced the statistical power of our 24-h ambulation experiments, preventing detection of potentially subtle changes in ambulation caused by the lynx1KO. It is appropriate to remark that the trends for decreased activity with lynx1KO were in the same direction as the significant changes seen for habituation as well as in functional assays in synaptosomal preparations.

### Nicotine-dependent behavior

**Fig 7:**
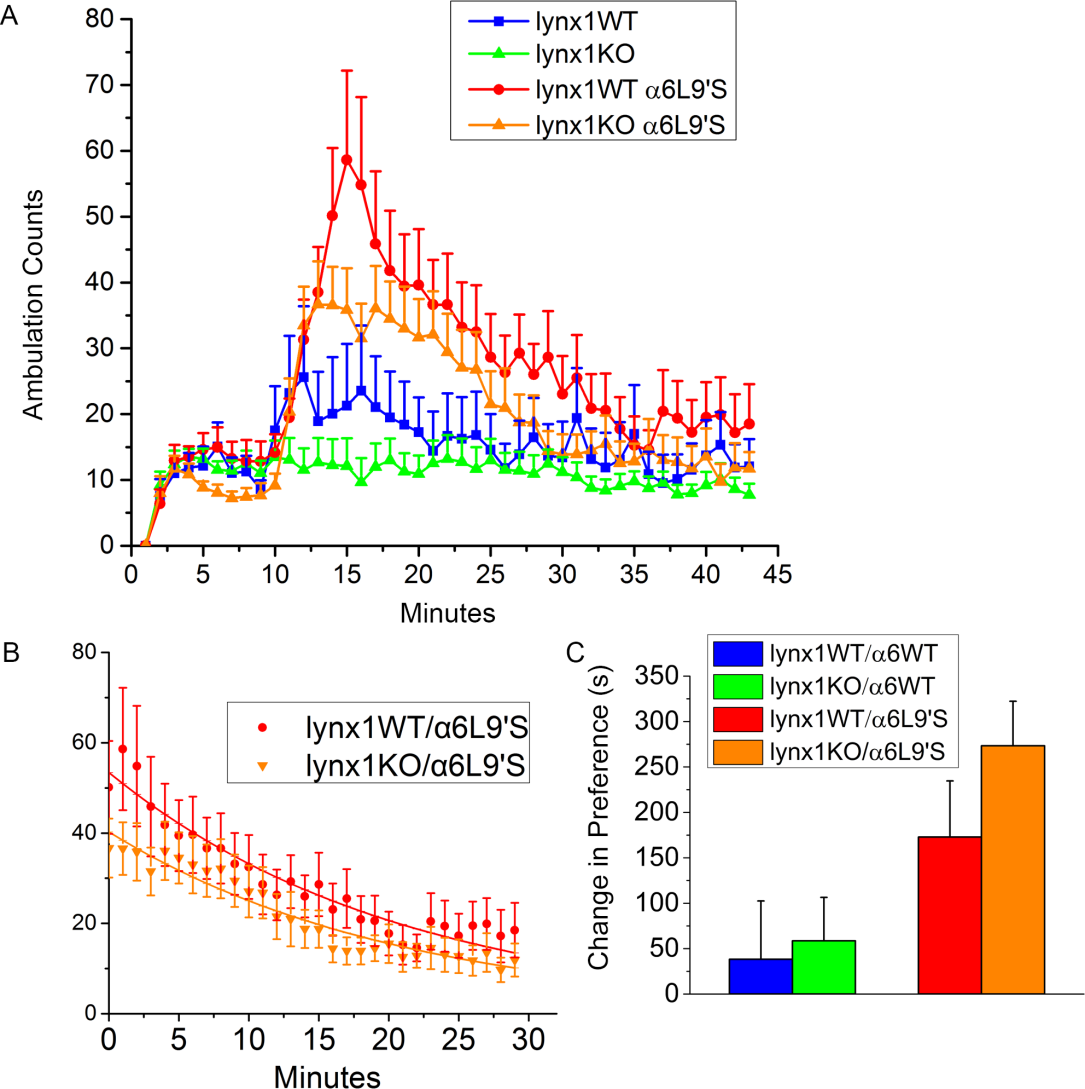
A) Response to a single injection of 0.15 mg/kg of nicotine. Animals were given a single IP injection of nicotine between minutes 8 and 9 of the experiment, and then their ambulations were measured until minute 43 of the experiment. Animal numbers: 15 lynx1WT/α6WT, 15 lynx1KO/α6WT, 16 lynx1WT/α6L9′S, 18 lynx1KO/α6L9′S. B) First-order exponential decay curves fitted to data for α6L9′S genotypes following nicotine injection. The lynx1KO significantly decreased peak counts (p value = <0.001) compared to lynx1WT without affecting time constant of decay. C) Average change in preference for mice undergoing CPP protocol with dose of 0.03 mg/kg of nicotine. Animal numbers: 10 lynx1WT/α6L9′S, 14 lynx1KO/α6L9′S, 12 lynx1WT/α6WT, 16 lynx1KO/α6WT). Both lynx1WT/α6L9′S and lynx1KO/α6L9′S genotypes showed a statistically significant response to nicotine (p value = 0.042 and <0.001, respectively, using a paired t-test). However, lynx1WT/α6L9′S and lynx1KO/α6L9′S were not significantly different from each other. The lynx1WT/α6WT and lynx1KO/α6WT genotypes did not show a significant CPP response, and were also not statistically different from each other.

Since a main question of this study was to determine whether lynx1KO might have effects on α6* nAChRs in the context of nicotine addiction, we tested whether nicotine had specific effects on lynx1KO animals. Previous studies in the α6L9′S mice established that these mice are hyperactive in response to a single dose of nicotine, with the largest response occurring for an injection of 0.15 mg/kg nicotine (free base) [21]. We injected the four groups of mice with nicotine (0.15 mg/kg, ip) at minute 8 and measured their response by ambulation (Fig 7A and 7B). We calculated a pre-nicotine baseline for each mouse as the mean ambulatory counts per minute from minutes 3 to 5, and a peak response to nicotine from minutes 14–16. Acute nicotine administration had little effect in either lynx1WT/α6WT (11.7 ± 2.6 ambulatory counts before nicotine vs 21.6 ± 9.1 peak counts after nicotine, t = 1.05, n = 15, ns) or lynx1KO/α6WT mice (13.0 ± 1.2 before vs 11.4 ± 4.0 after, t = 0.41, n = 15, ns). On the α6L9′S background the lynx1WT mice strongly responded to nicotine (13.7 ± 1.8 before vs 54.5 ± 12.1 after, t = 3.30, n = 16, p<0.01). The lynx1KO/α6L9′S mice also showed significant response to nicotine (10.5 ± 1.2 counts before vs 34.6 ± 5.6 counts after, t = 4.20, n = 18, p<0.001). A comparison of the two α6L9′S genotypes by t-test yields t = 1.50 with dof of 32, not statistically different.

Within this dataset, there were two groups of behavioral responses to acute nicotine injection. There were mice showing little response and those that became hyperactive in response to nicotine. Both types of responses were observed in all genotypes and both sexes, similar to the pattern noted for home cage activity. While relatively few mice with the α6WT background displayed locomotor activation calculated as a response of twice baseline, (3/15, 20% of lynx1WT/α6WT and 1/15, 7% of lynx1KO/α6WT), many mice on the α6L9′S background showed activation (10/16, 63% of lynx1WT/α6L9′S and 11/18, 61% of lynx1KO/α6L9′S). Comparison of peak activity for the responders on the α6L9′S background was 80.6 ± 13.6 counts for lynx1WT/α6L9′S (n = 10) and 50.9 ± 4.2 for the lynx1KO/α6L9′S group (n = 11) resulting in t = 2.08, a non-significant difference.

While the above analysis is complicated by the incomplete penetrance of the behavior in the α6L9′S genotypes, the lynx1 KO does have a strong tendency to decrease the response to nicotine in the responding group. Analyzing the data as first-order exponential decay curves allows all data points to be used, rather than just the peak 3 minutes, resulting in more statistical power (Fig 7B). This analysis provides two parameters, maximal response and a first-order rate of decay. For all the mice, we calculated parameters for maximal response of 53.4 ± 1.6 counts for lynx1WT/α6L9′S (n = 16) and 40.4 ± 1.2 for lynx1KO/α6L9′S (n = 18) with rate constants of 0.048 ± 0.003 min^-1^ and 0.048 ± 0.003 min^-1^, respectively. The lynx1KO significantly decreased the peak counts (t = 6.50, p<0.001) with no effect on rate of decay. For the responders only, analysis of the entire time course yielded maximal responses of 79.4 ± 2.5 counts for the lynx1WT/α6L9′S (n = 10) and 59.1 ± 2.2 for lynx1KO/α6L9′S (n = 11)(t = 6.10, p<0.001) with first-order rate constants of 0.052 ± 0.003 min^-1^ and 0.056 ± 0.004 min^-1^, respectively. Thus, while effects of the lynx1 deletion for the α6L9′S mice on ambulatory activity as mean counts/min in the peak 3 min for either the total population (t = 1.50, dof = 32) or the subset showing locomotor activation (t = 2.08, dof = 19) were not statistically significant, the effects of the lynx1KO for decreasing activity were highly significant when the complete data sets were analyzed by curve-fitting, as described here and above. The α6WT genotypes both had insignificant activation (see above), vitiating curve fits for these two genotypes.

Next, we asked whether lynx1 removal affects nicotine-mediated reward behavior as measured by conditioned place preference (CPP) (Fig 7C). We used a nicotine dose of 0.03 mg/kg, and we found that the lynx1WT/α6L9′S mice exhibited significant CPP with this dose of nicotine, as expected from results previously found for another nicotinic agonist [37]. Additionally, the lynx1KO/α6L9′S mice showed significant CPP with this dose of nicotine (*p* < 0.001 with paired t-test); however, no significant difference was found between lynx1WT/α6L9′S and lynx1KO/α6L9′S mice. Neither of the genotypes that lacked the α6L9′S mutation (lynx1WT/α6WT or lynx1KO/α6WT) developed CPP at this dose of nicotine. These results show that there is an effect of including the α6L9′S subunit in the genome, but no effect of lynx1 removal. Therefore it appears that lynx1 does not produce significant effects on this measure of nicotine-mediated reward via α6* nAChRs.

## Discussion

The data presented here show that the effects of removing lynx1 on α6* nAChRs are detectable but subtle, influencing α6* nAChR activity, DA release, and some locomotor behaviors. Lynx1 can associate with α6YFPβ2 when transiently overexpressed in cell lines (Fig 1), but the addition of lynx1 did not affect the response to nicotine in transfected cells (Fig 2). Because functional studies in neurons may be more appropriate than in clonal cell lines, we subsequently used the lynx1KO and α6L9′S mouse models to study the effects of lynx1 on α6* nAChR. Since these receptors are normally present in only a few brain regions, we studied whether those brain regions exhibited any changes when lynx1 was deleted.

As the Introduction notes, the strategy of generating mice with hypersensitive nAChRs enables investigation of behavioral traits at nicotine doses that activate only the hypersensitive subtypes as well as more precise biochemical and physiological assays. Previously, the hypersensitive mouse strategy has been applied to α2*-, α4*-, α6*-, α7*-, and α9*-nAChRs via M2 mutations in these α subunits, and has allowed researchers to assign nicotine-induced behavioral, biochemical, and physiological phenotypes to these nAChR subtypes [23]. However, one should note that quantitative aspects of our conclusions depend to some extent on the high levels of α6* nAChR activity in the α6L9’S mice.

In synaptosomal preparations from mice, we discerned that lynx1KO reduced the amount of ^86^Rb^+^ efflux in the SC that was mediated through α6* nAChRs, suggesting that there were fewer or functionally altered nAChRs when lynx1 was absent. These data indicate that lynx1 is necessary for the normal function of α6*nAChRs. This decrease in activity of α6* nAChRs was detectable in α6WT only in SC. However, in the α6L9′S mice with a larger α6 component, the nicotine-induced DA release in ST and OT as well as the ^86^Rb^+^ efflux in SC all followed this pattern. In addition to these biochemical measures, absence of lynx1 decreases the ambulation of the α6L9′S mice in a novel environment, as well as activation in response to an injection of nicotine. Our binding data provide evidence that the lynx1-induced changes may not be simply a decrease in α6* nAChR numbers as no changes were seen in numbers of α6β2 binding sites across 10 regions. Binding data include both surface-expressed sites and intracellular sites, so it is possible that lynx1 changes the ratio between these two classes. Further, there might be lynx1-induced alteration in the relative numbers of subtypes of α6* nicotinic receptors. It has been shown that in the absence of the α4 subunit, the α6* mediated DA release as well as the hyperactive behavior of α6L9′S mice is decreased [27]. The (α4/β2)(α6L9′S/β2)(β3) form of nicotinic receptors (where / denotes an agonist-binding interface) is absent in these less active α4KO/α6L9’S mice [27]. Therefore, instead of changing numbers of surface-expressed α6* nicotinic receptors, lynx1 could function by decreasing the ratio of (α4/β2)(α6L9′S/β2)(β3) to (α6L9′S/β2)_2_(β3). It has been shown that lynx1 can alter the ratio of stoichiometries of α4β2* nicotinic receptors [17].

Neither patch-clamp electrophysiology of DA neurons in the SNc, nor FSCV in slices of the striatum detected any effect of lynx1 removal, while Rb^+^ efflux in SC and DA release in ST and OT (both synaptosomal preparations) did show effects of lynx1 on function of α6* nicotinic receptors. There are several possible explanations for this apparent inconsistency. In comparing the SNc to the striatum (sites of patch clamp vs synaptosomal DA release), lynx1 may have differential effects on nicotinic receptors that are localized on the cell body versus the terminals of these neurons [35]. Additionally, lynx1 may be necessary for normal targeting of the α6* nAChRs to the DA terminals, causing differences in the terminals that are undetectable when recordings are done from the cell bodies. To try to address the possibility of differences between the terminals and cell bodies, we used FSCV in the dorsal striatum as an alternative measure of DA release. In agreement with previously published data, we measured a significant increase in the size of the response from 1p to 4p in lynx1WT/α6L9′S compared to lynx1WT/α6WT mice [25]. The addition of α-CtxMII reduced DA release by ~ 90% in lynx1WT/α6L9′S, far greater than previous reports in WT mice, but consistent with previous reports in the α6L9′S mice [25]. However, we measured no differences between lynx1KO/α6L9′S mice and lynx1WT/α6L9′S mice in the presence of α-CtxMII. While both the FSCV and synaptosomal DA release experiments were conducted using striatal tissues, the FSCV is electrically stimulated, while the synaptosome experiments measured nicotine-induced release. Electrical stimulation activates multiple transmitter systems that contribute to the sum of the DA release response, while nicotine activates only nAChRs, possibly leading to different results. The synaptosomal nicotine-evoked DA release is also concentration-dependent. If lynx1 has changed the ratio of subtypes of α6* nAChRs, a functional change would be more readily measured by concentration-response assays, where differences among receptor subtypes with altered potency and efficacy may be detected.

Previous studies have shown that lynx1 acts as a brake or a negative modulator of some nicotinic receptor subtypes, including α4β2, α3β4, α5α3β4, and α7 nAChRs [11–13, 16]. However, the present data present a contrasting case with α6* nAChRs: lynx1 normally augments the function of α6* nAChRs, and removing lynx1 actually dampens the activity of both α6 and α6L9’S nAChRs. This was unexpected and indicates that the α6 nAChR subunit likely has an atypical interaction with lynx1, perhaps like the lynx1 paralog Ly6g6e which increases α4β2 responses [38]. Most of the significant effects of lynx1 KO on α6* nAChRs were measured using the α6L9′S mutation which could indicate some difference in the interaction of lynx1 with α6WT vs α6L9′S. However, in the SC a significant effect in the same direction was seen with α6WT.

Another unanticipated finding was the lack of effect of lynx1 on non-α6* nAChRs in the brain regions currently under investigation. Based on previous findings, lynx1 removal might be expected to have effects in the α-CtxMII-resistant populations of nicotinic receptors in the DA neurons, which include α4β2 and α5α4β2 [7, 39]. However, we observed no effects of lynx1 on α-CtxMII-resistant populations, in synaptosomal preparations, electrophysiology, or FSCV experiments. The α4β2* nAChR population of DA neurons is highly enriched for the α5α4β2 subtype [40]. If the major effect of lynx1 on α4β2* is to selectively decrease the “high-sensitivity” or HS form, (α4β2)_2_β2 [17], the population in DA terminals with large amounts of another HS form, (α4β2)_2_α5, could be less affected by lynx1. With current techniques, it is not possible to distinguish between functions of the two HS forms in striatal samples. In addition, the DA release assay used here measures only the HS forms of α4β2* nicotinic receptor, and therefore would not detect any changes in “low-sensitivity” forms.

Many tobacco dependent people find smoking rewarding, and decoupling reward from dependence will be an important variable in smoking cessation strategies. The lynx1WT/α6L9′S mice show CPP, a reward-related behavior, at strikingly low doses of agonist [37], but full dose-response relations for CPP have not been studied in this strain. The present experiments were performed at one nicotine dose, 0.03 mg/kg, and showed robust CPP with no effects of lynx1 deletion. It remains possible that lower doses of nicotine would reveal an effect of lynx1 deletion.

In summary, we have found that α6* nAChRs are modulated by lynx1. This represents a new aspect of regulation for this subclass of nicotinic receptors. While we found no connection between lynx1 and nicotine CPP at the nicotine dose used, the finding that lynx1 modulates α6* nAChR-dependent locomotor activity and neurotransmitter release may be helpful in understanding some aspects of addiction to smoking. Further, since α6* nAChRs are involved in both motor function and reward, our understanding of the selective functional and behavioral actions of lynx on α6* nAChRs could be useful for addressing motor abnormalities and dyskinesias, without risk of confounding abuse liabilities.

## Materials and methods

### Cell culture, western blot, and co-immunoprecipitation

HEK293 and Neuro2a (N2a) cells were obtained from ATCC and were maintained with DMEM, Na pyruvate, Pen Strep antibiotics (Thermo Fisher), and 10% FBS (HEK293) or 45% DMEM, 45% Optimem, 10% FBS, and Pen Strep antibiotics (N2a). Cells were transfected using ExpressFect (Denville Scientific) and plasmids pCI-neo-α4GFP, pCI-neo-α6YFP, pCI-neo-β2WT, and pc-DNA3.1-lynx1. For the co-immunoprecipitation, HEK293 cells were transfected; 48 h post transfection, cells were harvested by scraping with PBS, followed by centrifugation for 4 minutes at 4000 rpm (1300 x g) (Eppendorf 5415C, Hauppauge, NY). Cells were lysed using ice-cold extraction buffer (50 mM Tris pH 7.4, 50 mM NaCl, 1% NP40, 1 mM EDTA, 1 mM EGTA, supplemented with 1% P8340, and 4 mM PMSF). The cells were triturated by pipetting 20–30 times in the extraction buffer and then allowed rest on ice for 5–10 min. Following that, they were centrifuged at 14,000 rpm (16,000 x g) (Eppendorf 5415C, Hauppauge, NY) for 5 min at 4°C. The supernatant was transferred to a new tube; 50 μl was set aside for the input lane.

To bind the antibody to the protein A Dynabeads (Invitrogen, Carlsbad, CA; cat # 11122), 5 μg of antibody was diluted into 200 μl PBS with 0.02% Tween-20, mixed with 50 μl of beads for 20 minutes at room temperature and then washed once with PBS containing 0.02% Tween-20. The cell supernatant was mixed with the antibody bound beads for 1 h at room temperature. Subsequently, the beads were washed 3x with PBS, then heated to 70°C for 10 minutes in 20 μL of 1x Laemmli buffer (Bio-Rad Laboratories, Hercules, CA).

The samples were loaded onto a 4–10% gradient gel (Bio-Rad) and electrophoresed for 1.5 h at 100 V. The protein was transferred to nitrocellulose membrane using a semi-dry transfer system for 15 min at 15 V. The membrane was blocked with 5% milk for 1 h, then probed with primary goat anti-lynx1 antibody (Santa Cruz Biotechnology, Inc, Santa Cruz, CA) at 1:500 in 5% milk overnight at 4°C or rabbit anti-GFP (Invitrogen) antibody at 1:500 overnight. The secondary antibodies used were goat anti-rabbit at 1:5000 for one h and donkey anti-goat at 1:2000 for two h. Western blots were imaged either using anti-HRP secondary antibodies and film, or using fluorescent secondary antibodies and a LI-COR (Lincoln, NE 68504) Odyssey imaging system.

### Cell electrophysiology

Cells were maintained and transfected as described above, but for electrophysiology they were plated at a lower density onto glass coverslips. 48 h post-transfection, the cells were transferred to the 32°C recording chamber, where they were perfused with oxygenated (95% O_2_ / 5% CO_2_) ACSF. The ACSF consists of (in mM): 124 NaCl, 3 KCl, 1.25 NaH_2_PO_4_, 26 NaHCO_3_, 10 glucose, 1.3 MgSO_4_, and 2.5 CaCl_2_ [41]. Cells were visualized with an Hg lamp to determine which had been transfected with α6GFP, and a whole cell patch clamp configuration was obtained. Cells were puffed with nicotine for 200 ms using a Picospritzer (Parker Hannafin)

### Animals

All animal experiments were conducted in accordance with the *Guide for Care and Use of Laboratory Animals*, and protocols were approved by the Institutional Animal Care and Use Committee at Caltech (Protocol 1386–13) or the University of Colorado at Boulder. Mice used for experiments were generated from breeding pairs where both parents were heterozygous for the lynx1KO allele and one of the parents had the BAC transgene containing the α6L9′S mutation from line 2 (copy number 18.9±0.9, Cohen et al, 2012) [13, 21]. Animals were group housed, except for immediately before and during behavioral experiments. The animals had free access to food and water and were on a 13 h dark: 11 h light cycle. When conditions allowed, mice were used for novel environment experiments, then AMBA, and finally for single injection of nicotine. Mice of both sexes were studied. We noted no marked differences between males and females; therefore we conducted no systematic studies on this point.

### RNA-Seq materials and methods

For laser capture microdissection, C57BL6/DBA WT mice were deeply anesthetized with sodium pentobarbital (100 mg/kg; i.p.) and sacrificed by decapitation. Our methods pipeline for fabricating cDNA libraries via laser capture microdissection, and performing RNA-Seq were previously described [42]. Briefly, whole brains from 4 month old male mice (*n = 3*) were collected (post-mortem interval of < 5 min), fresh frozen over dry ice, and stored at -80°C. Midbrain cryostat [43] sections (20 μm) were mounted on UV-treated Zeiss Membrane Slides (1.0 PEN NF), air dried for 5 minutes, and stained with cresyl violet for 1 minute. The sections were rinsed, dried and then visualized under brightfield illumination at 400X magnification on a Zeiss PALM Laser Capture Micro Dissection microscope. Twenty putative DA^+^ cell bodies from the SNc were dissected using multiple low laser energy pulses, and were catapulted into Zeiss 200 μL adhesive caps. Cell lysis solution (Illumina, San Diego, CA) containing 3′ SMART reverse transcription primers and quantitation controls (“spikes”) were then added into the pool of cells prior to freezing.

To fabricate cDNA libraries, we prepared amplified cDNA from RNA, using Clontech's SMARTer™ Ultra Low RNA system for Illumina Sequencing (Clontech, Mountain View, CA) as previously described [44]. Poly(A)^+^ RNA was reverse transcribed through oligo dT priming to generate full-length cDNA, which was then amplified using 22 cycles, using Clontech's Advantage 2 PCR system. RNA-Seq libraries were constructed using the Nextera DNA Sample Prep kit (Illumina). cDNA was “tagmentated” at 55°C with Nextera transposase, and tagmented DNA was purified using Agencourt AMPure XP beads (Beckman Coulter Genomics). Purified DNA was amplified using five cycles of Nextera PCR. After quality control measures of yield and fragment length distribution were taken using the Qubit fluorometer (Invitrogen, Carlsbad, CA) and the Agilent (Santa Clara, CA) Bioanalyzer, 50 bp or 100 bp sequencing reads were generated on the Illumina HiSeq instrument. Each sequencing library generated > 20 million uniquely mapping reads.

For computational analysis, 50 bp or 100 bp sequence tags were mapped to the mouse genome using TopHat 1.3.2 [45]. We quantified transcript abundance (FPKM: fragments per kilobase per million mapped reads (expression values)) using Cufflinks. We annotated the transcripts with genome annotations provided by ENSEMBL. Data were analyzed and graphs were generated using GraphPad Prism 5.

### Rubidium efflux

Previously described methods [46] were used to measure ^86^Rb^+^ efflux from synaptosomal preparations of mouse brain regions using carrier-free ^86^RbCl purchased from Perkin Elmer Life Sciences (Boston, MA). Aliquots of the synaptosomal preparation, loaded with ^86^Rb^+^, were superfused at 2.5 ml/min. A 5-s exposure to nicotine stimulated efflux; sample effluent was pumped through a 200 μl flow-through Cherenkov cell in a β-RAM Radioactivity HPLC detector (IN/US Systems, Inc., Tampa, Fl.) allowing continuous monitoring. Stimulated levels of ^86^Rb^+^ efflux as units were determined as evoked cpm exceeding baseline level of efflux, summed and normalized to baseline level.

### DA release

Previously described methods [27] were followed using 7,8-[^3^H]DA (20–40 Ci/mmol) obtained from Perkin Elmer Life Sciences (Boston, MA). Crude synaptosomal preparations from freshly dissected brain regions were allowed to take up tracer [^3^H]DA prior to superfusion at 0.7 ml/min for 10 min. Release of DA was stimulated by exposure to nicotine for 20s. Parallel aliquots were exposed to 50 nM α-conotoxin MII (α-CtxMII) (generously provided by Dr. J. Michael McIntosh, University of Utah) for 5 min before the nicotine exposure. Fractions (10 s, ~0.1 ml) were collected into 96-well plates using an FC204 fraction collector (Gilson, Inc., Middleton, WI) for 3.8 min starting one min before nicotine exposure. DA release units are calculated as evoked cpm exceeding baseline cpm, summed and normalized to baseline cpm.

### Membrane binding

Membrane binding experiments were conducted on tissue remaining from synaptosomal experiments after a lysis step and further washing by resuspension and centrifugation using the methods of Whiteaker et al. [8]. [^125^I]epibatidine (2200 Ci/mmol purchased from Perkin Elmer Life Sciences (Boston, MA)) was used at 200 pM with a 2 h incubation at room temperature. Additions to parallel samples included 50 nM cytisine (to isolate the cytisine-sensitive population in 6 brain regions), 50 nM α-CtxMII (to isolate the α-CtxMII-sensitive population in 3 brain regions) or 100 μM nicotine for blank determination. Data are calculated as fmol bound/ mg protein and expressed as % of lynx1WT/α6WT genotype.

### Autoradiography

For binding experiments using autoradiography, published methods were followed [8, 47, 48]. [^125^I]epibatidine with unlabeled 6-I-epibatidine (kindly donated by Dr Kenneth Kellar, Georgetown University) (total 200 pM) with or without αCtxMII (50 nM) was used to determine levels of nicotinic receptor with and without α6β2 sites. [^125^I]epibatidine with and without cytisine (50 nM) was used to determine α4β2 sites. Blanks were equal to film background. After incubation (4 h, rt), washing and drying steps, slides were exposed to Packard Super Resolution Cyclone Storage Phosphor Screens (Perkin Elmer Life Sciences, Boston, MA) for subsequent quantitation compared to tissue paste standards using Optiquant software (Perkin Elmer Life Sciences). Ten brain regions known to express both sites were quantitated for the Lynx1WT and KO on the α6L9′S genotype. Data are expressed as cpm/mg wet lynx1WT/α6WT.

### Slice electrophysiology

Mice used for midbrain recordings were ages P17 to P25. All animals were genotyped before and after the experiment (animal numbers: 11 lynx1WT/α6L9′S, 13 lynx1KO/α6L9′S, 8 lynx1WT/α6WT, 8 lynx1KO/α6WT). Animals are euthanized with CO_2_ gas, then subjected to cardiac perfusion with an oxygenated (95% O_2_ / 5% CO_2_) ice-cold glycerol-substituted ACSF (in mM: 250 glycerol, 2.5 KCl, 1.2 NaH_2_PO_4_, 26 NaHCO_3_, 11 glucose, 1.3 MgCl_2_, and 2.4 CaCl_2_). Each animal was then decapitated; the brain dissected and mounted on a vibratome in ice-cold glycerol ACSF. 250 μM coronal sections were made using a vibratome (DTK-1000; Ted Pella, Redding, CA). Slices were allowed to recover for 1 h in regular ACSF bubbled with 95% O_2_ / 5% CO_2_ at 32°C, then warmed to room temperature. The ACSF consisted of (in mM): 124 NaCl, 3 KCl, 1.25 NaH_2_PO_4_, 26 NaHCO_3_, 10 glucose, 1.3 MgSO_4_, and 2.5 CaCl_2_ [41]. After 15 min at room temperature the slices were put into fresh room temperature ACSF. Recordings were made in a chamber perfused with ACSF at 32°C, bubbled with 95% O_2_ / 5% CO_2_, at a rate of 1–2 mL/min. The internal pipette solution in mM consisted of 135 K gluconate, 5 EGTA, 10 HEPES, 2 MgCl_2_, 0.5 CaCl2, 3 Mg-ATP, and 0.2 GTP. The slices were visualized with an upright microscope (BX50WI, Olympus) and near-infrared illumination. Recordings were made from the VTA or SNc, and a picture was taken of each cell recorded from to verify location. We tested for I_h_ and measured the firing rate in each cell to determine that we were indeed recording from a DA neuron. Patch pipettes were made using a programmable microelectrode puller (P-87; Sutter Instrument Co., Novato, CA) and pipette resistances were 4–8 MΩ. Recordings were made with an Axon Multiclamp 700A Amplifier and recorded using Clampex 10, both from Molecular Devices Axon (Sunnyvale, CA). Data were sampled at 5 kHz and low-pass filtered at 2 kHz. The holding potential was -65 mV. Over a period of 1.4 s, the puffer pipette was moved to within one cell length of the cell by a piezoelectric controller (Burleigh Instruments; Fishers Park, NY). There was a 100 ms pause; a puff of 200 ms drug was applied using a picospritzer; and then the puffer pipette was retracted over 360 ms. Data were analyzed using Clampfit 10, also from Molecular Devices.

### Fast-scan cyclic voltammetry (FSCV)

Electrodes were fabricated using carbon fiber (7 μM, unsized from Goodfellow) and glass without a filament from Sutter. One carbon fiber was pulled through a glass micropipette. This was then pulled into two electrodes on a Sutter P-87 puller. The carbon fibers were trimmed, and the electrodes dipped into epoxy for 7 min and then quickly rinsed in acetone. Electrodes were baked overnight at 80°C to cure the epoxy. The carbon fiber was trimmed to an appropriate length just before use. The carbon fiber was placed in the dorsal striatum, just below the surface of the slice. The animals used in these experiments were 18–27 weeks old (Animal numbers: 10 lynx1WT/α6L9′S, 7 lynx1KO/α6L9′S). Slices were prepared as for electrophysiology, except the slices were 300 μM thick and were taken from the striatum. Recordings were made with an Axon Multiclamp 700B Amplifier and recorded using Clampex 9, both from Molecular Devices Axon (Sunnyvale, CA). Voltage ramps of 20 ms duration were applied to the carbon fiber at 100 ms intervals (sampling interval 20 μs). After the current waveform stabilized, a pulse was applied to an adjacent region of the dorsal striatum using a bipolar stimulating electrode (FHC). The pulse was sufficient to elicit maximal stimulation, and the 2p and 4p stimuli were delivered at 100 Hz. The peak response was measured, and a single exponential fit was used to determine tau. See [49, 50]. The electrodes were calibrated at the end of each experiment with 1 μM DA.

### Spontaneous activity in novel environment

Mice used in the study of locomotion were eight to sixteen weeks old by the beginning of the experiment. Horizontal locomotor activity was measured with an infrared photobeam activity cage system (San Diego Instruments; San Diego, CA). Ambulation events were recorded when two neighboring photobeams were broken in succession. Mice were moved to the room immediately before the experiment and put into fresh cages at the start of the novel environment test. Their activity was measured for 33 min. Mice were returned to their home cages following the experimental period (Animal numbers: 24 lynx1WT/α6L9′S, 14 lynx1KO/α6L9′S, 20 lynx1WT/α6WT, 18 lynx1KO/α6WT).

### Automated mouse behavior analysis (AMBA)

Video-based software analysis of home cage behavior was conducted as described previously [27, 51]. Mice that were normally group housed were singly caged and habituated to the video recording room for 24 h before recording (animal numbers: 24 lynx1WT/α6L9′S, 20 lynx1KO/α6L9′S, 17 lynx1WT/α6WT, and 17 lynx1KO/α6WT). The video recording began the following day (2 h before the dark phase) and continued for 23.5–24.0 h, using dim red lights for recording during the dark phase. The videos were analyzed using the definitions and settings described in HomeCageScan 3.0 software (CleverSys).

### Locomotion in response to nicotine injections

Acute locomotor activity in response to nicotine was measured by recording ambulation events for 43 min. This was recorded with the same equipment as the spontaneous activity assay. Groups of eight mice were singly housed in clean cages and their baseline level of activity was recorded for eight min (animal numbers: 16 lynx1WT/α6L9′S, 18 lynx1KO/α6L9′S, 15 lynx1WT/α6WT, 15 lynx1KO/α6WT). Mice were removed from their cage, injected with nicotine 0.15 mg/kg intraperitoneally and returned to the cage within 30 sec.

### Conditioned place preference (CPP)

The conditioned place preference test apparatus consists of a three-chamber rectangular cage with a center neutral gray compartment (Med Associates, Inc., St. Albans, VT). One test compartment is black with a stainless-steel grid rod floor. The second test chamber is white with a square stainless-steel mesh floor. Guillotine doors separate the chambers and can be fixed in the closed or opened position (Animal numbers: 10 lynx1WT/α6L9′S, 14 lynx1KO/α6L9′S, 12 l lynx1WT/α6WT, 16 lynx1KO/α6WT).

The day before the test, the animals are moved into the room with the CPP apparatus and singly housed in clean cages. The CPP protocol was a 10-day experiment. On day one (pre-test day) a mouse was placed in the central compartment and allowed free access to all chambers. The time spent in each chamber was recorded over a 20 min period. Days 2–9 were training days. Intraperitoneal injections of nicotine free base (0.03 mg/kg) were paired with one of the conditioning chambers, while injections of saline are paired with the other. In a biased design, mice received nicotine in the less preferred chamber as determined on day one of the experiment. Conditioning trials for the nicotine-associated chamber occurred on days 2, 4, 6, and 8; conditioning trials for the saline-associated chamber occurred on days 3, 5, 7, and 9. Each training trial lasted 20 min. On the last day (post-test) of the experiment, the mouse was once again given free access to all chambers for 20 min. The time spent in each chamber during the pre-test was subtracted from the time spent in each chamber on the post-test day. A preference toward the nicotine-associated chamber compared to baseline is a measure of the reward behavior associated with nicotine [22].

### Statistics

[^125^I]epibatidine binding, ^86^Rb^+^ efflux, and nicotine-mediated DA release were analyzed for effect of lynx1KO within each α6 genotype by t-test, For the electrophysiology a Kruskal-Wallis ANOVA with post-hoc Dunn’s test was used. FSCV data was analyzed using a rank-sum test. For the habituation and nicotine-mediated ambulation, a two-tailed t-test was used to make comparisons between the two groups that had the same α6 genotype. The AMBA data were analyzed using a Fisher’s exact test to compare the high and low activity groups with the effect of lynx1 on each α6 genotype. The CPP data were analyzed using a paired t-test for before and after training. All error bars represent the SEM.
